# Case report: Neuronal intranuclear inclusion disease initially mimicking reversible cerebral vasoconstriction syndrome: serial neuroimaging findings during an 11-year follow-up

**DOI:** 10.3389/fneur.2024.1347646

**Published:** 2024-02-09

**Authors:** Gha-Hyun Lee, Eugene Jung, Na-Yeon Jung, Takeshi Mizuguchi, Naomichi Matsumoto, Eun-Joo Kim

**Affiliations:** ^1^Department of Neurology, Pusan National University Hospital, Pusan National University School of Medicine and Medical Research Institute, Pusan, Republic of Korea; ^2^Department of Neurology, Pusan National University Yangsan Hospital, Pusan National University School of Medicine and Medical Research Institute, Yangsan, Republic of Korea; ^3^Department of Human Genetics, Yokohama City University Graduate School of Medicine, Yokohama, Japan

**Keywords:** NIID, neuronal intranuclear inclusion disease, perfusion, MRI, episodic neurological dysfunction

## Abstract

Neuronal intranuclear inclusion disease (NIID) is a rare, progressive neurodegenerative disorder known for its diverse clinical manifestations. Although episodic neurogenic events can be associated with NIID, no reported cases have demonstrated concurrent clinical features or MRI findings resembling reversible cerebral vasoconstriction syndrome (RCVS). Here, we present the inaugural case of an adult-onset NIID patient who initially displayed symptoms reminiscent of RCVS. The 59-year-old male patient’s initial presentation included a thunderclap headache, right visual field deficit, and confusion. Although his brain MRI appeared normal, MR angiography unveiled left posterior cerebral artery occlusion, subsequently followed by recanalization, culminating in an RCVS diagnosis. Over an 11-year period, the patient encountered 10 additional episodes, each escalating in duration and intensity, accompanied by seizures. Simultaneously, cognitive impairment progressed. Genetic testing for NIID revealed an abnormal expansion of GGC repeats in *NOTCH2NLC*, with a count of 115 (normal range, <60), and this patient was diagnosed with NIID. Our report highlights that NIID can clinically and radiologically mimic RCVS. Therefore, in the differential diagnosis of RCVS, particularly in cases with atypical features or recurrent episodes, consideration of NIID is warranted. Additionally, the longitudinal neuroimaging findings provided the course of NIID over an 11-year follow-up period.

## 1 Introduction

Neuronal intranuclear inclusion disease (NIID) is a rare progressive neurodegenerative disorder characterized by eosinophilic hyaline intranuclear inclusions in the central and peripheral nervous systems and various visceral organs (1). Diagnosis of NIID can be challenging owing to its highly heterogeneous clinical symptoms, which include cognitive impairment, tremor, peripheral neuropathy, and autonomic dysfunction. Although hyperintensity along the corticomedullary junction (CMJ) in diffusion-weighted imaging (DWI) on brain magnetic resonance imaging (MRI) can facilitate the diagnosis of NIID, it can easily be missed when patients do not show this characteristic MRI finding.

Reversible cerebral vasoconstriction syndrome (RCVS) is a group of conditions characterized by the reversible narrowing of the cerebral arteries associated with thunderclap headaches and is sometimes accompanied by neurological deficits. While there have been several reports of episodic neurogenic events in NIID (2, 3), no case of NIID accompanied by the clinical features or MRI findings of RCVS has been reported. This report describes a patient presenting with headache and neurological deficits accompanied by cerebral vasospasm similar to RCVS that finally turned out to be NIID after an 11-year follow-up.

## 2 Case description

**Figure 1:**
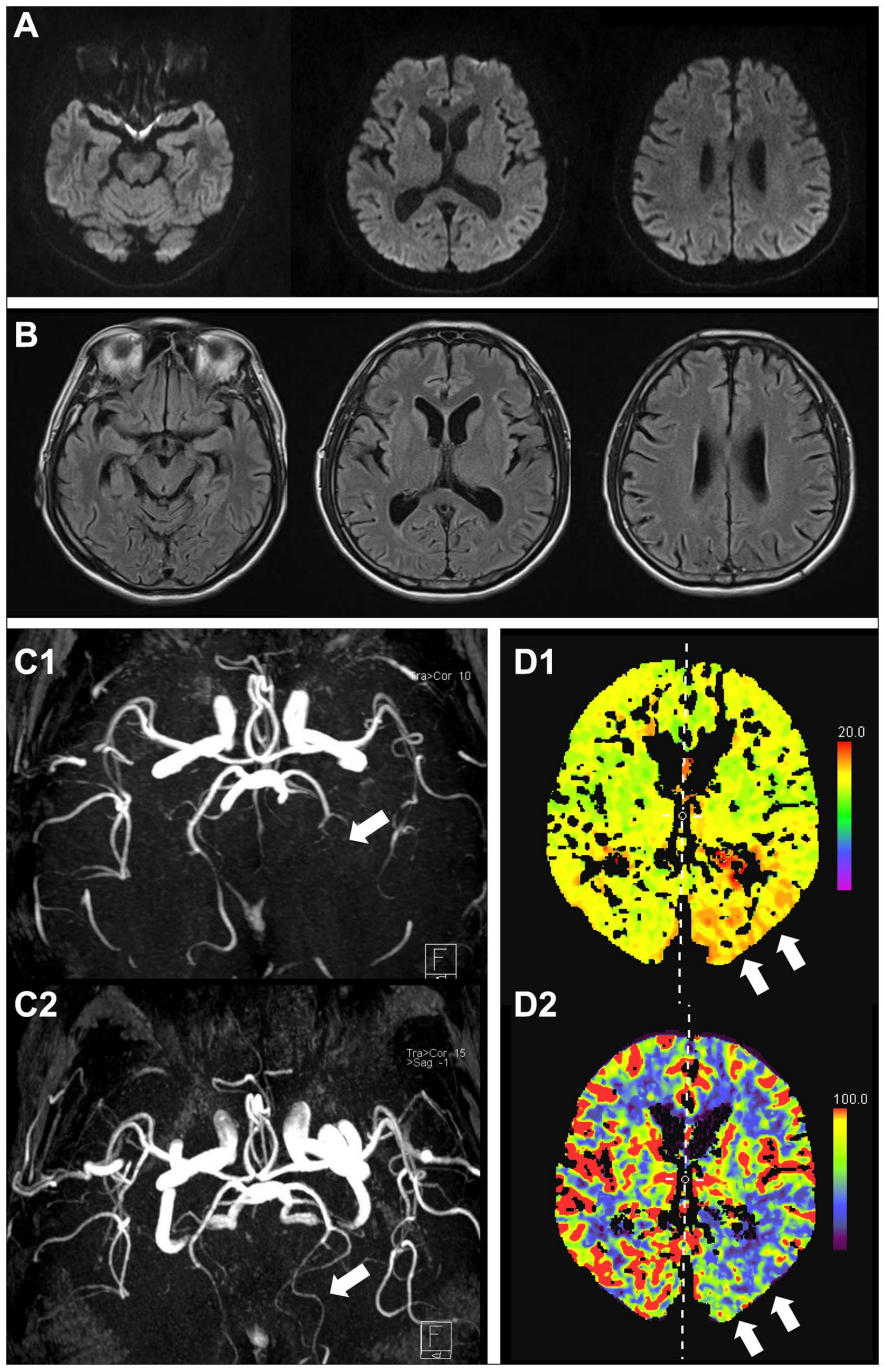
Brain MRI at initial onset showing no apparent lesions (**A**, DWI; **B**, FLAIR). MRA shows occlusion of the left posterior cerebral artery (**C1**, arrow), which was recanalized at the next follow-up 6 months later (**C2**, arrow). CT perfusion images showing a delay in the time-to-peak map (**D1**, arrows) and reduced cerebral blood flow (**D2**, arrows) in the left posterior cerebral artery. MRI, magnetic resonance imaging; MRA, magnetic resonance angiography; CT, computed tomography; DWI, diffusion-weighted image; FLAIR, fluid-attenuated inversion recovery.

A 59-year-old man presented with an acute onset of severe thunderclap headache in the right frontotemporal region, accompanied by nausea, vomiting, right visual field deficit, and confusion. The patient had a history of similar recurring symptoms since his early 50s, which were relieved within an hour and occurred once a year. Initial laboratory tests, including cerebrospinal fluid and cytological, biochemical, and microbiological examinations, were unremarkable. DWI and fluid-attenuated inversion recovery (FLAIR) sequences performed on the day the headache started were normal (Figures 1A,B). However, magnetic resonance angiography (MRA) revealed occlusion of the left posterior cerebral artery (PCA) (Figure 1C1). Computed tomography (CT) perfusion revealed a delay in the time-to-peak map and reduced cerebral blood flow in the left occipital lobe (Figure 1D). He was administered nimodipine, and his symptoms resolved within 1 day. Follow-up MRA 6 months later revealed recanalization of the occluded left PCA (Figure 1C2). Based on clinical features and the course, the patient was diagnosed with RCVS.

**Figure 2:**
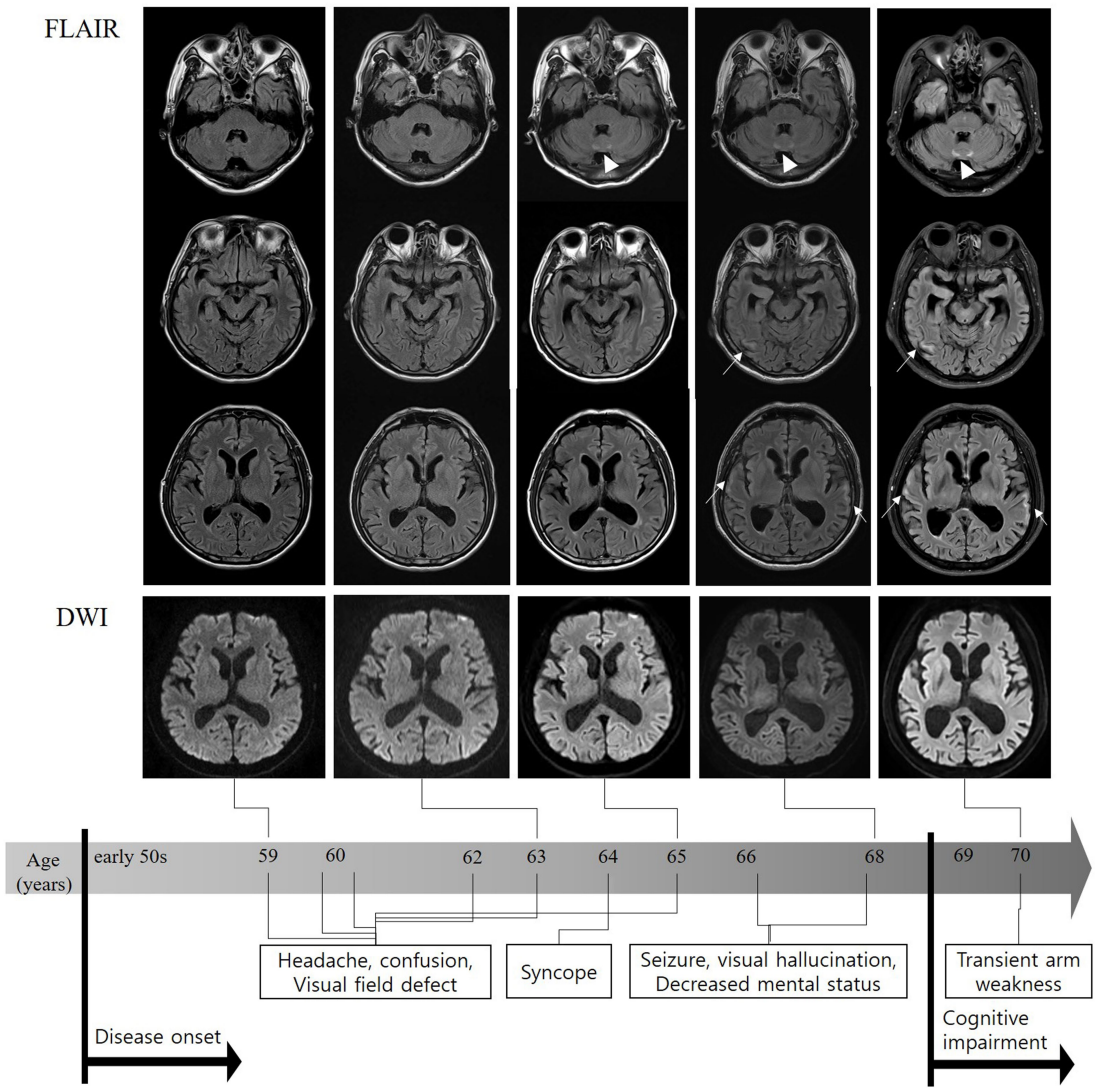
FLAIR sequences on MRI performed 10 years after onset showing diffuse brain atrophy, particularly in the bilateral temporal lobes and subtle high signal intensities in the bilateral temporal corticomedullary junction (arrows). Paravertebral high-intensity signal changes were observed a few years earlier than cortical changes (triangles). No abnormally high signal intensity was observed on diffusion-weighted MRI. FLAIR, fluid-attenuated inversion recovery; MRI, magnetic resonance imaging.

The patient experienced 10 more episodes over the course of 11 years, with episodes becoming longer and more severe over time (Supplementary Table S1). DWI at each episode revealed no abnormalities; however, MRA and CT perfusion demonstrated variable findings, ranging from normal to reduced blood flow in the right or left parietal–temporal lobe (Figure 2). In two episodes at the ages of 66 and 68 years, the patient experienced seizures and hallucinations lasting for more than 2 weeks. Video-electroencephalography (EEG) monitoring revealed continuous slowing in the left hemisphere without epileptiform discharges. All autoimmune laboratory test results were normal. Genetic tests for mitochondrial encephalopathy, lactic acidosis, and stroke-like episodes (MELAS) and myoclonic epilepsy with ragged red fibers were negative. His symptoms gradually improved after steroid pulse therapy and the administration of anti-seizure medication. At the age of 67, his memory began to decline. At 69, approximately 10 years after his initial presentation, he was hospitalized for evaluation of progressive cognitive decline. The patient had a family history of dementia (mother and three paternal aunts). Neuropsychological examination revealed moderate to severe deficits in all cognitive domains (Mini-Mental State Examination score, 24/30; Clinical dementia rating, 1). Postural/active upper limb tremors were observed; however, no other significant findings were observed. EEG showed intermittent generalized slow waves, and FLAIR images showed diffuse cortical atrophy with multifocal hyperintensities in the CMJ of the bilateral frontal, temporal, and parietal areas and the paravermis. However, no abnormally high signal intensity was observed on DWI (Figure 2). Tests for other neurodegenerative diseases, such as cerebrospinal fluid real-time quaking-induced conversion (CSF RT-QulC) assay for detecting pathogenic prion protein (PrP^sc^) for Creutzfeldt–Jakob disease and Amyloid PET scan for Alzheimer’s disease (AD), returned negative results. Based on progressive cognitive deficits and tremors, which are atypical clinical features of RCVS, and paravermal hyperintensity on FLAIR, which is one of the imaging markers of NIID, a genetic test for NIID was conducted, revealing an abnormal expansion of GGC repeats in *NOTCH2NLC*, with a count of 115 (normal range, <60) (Supplementary Figure S1). Since there is no disease-modifying therapy for NIID, the patient is prescribed levetiracetam and propranolol to manage seizures and tremors, with regular outpatient visits scheduled quarterly.

## 3 Discussion

The patient was initially diagnosed with RCVS based on reversible thunderclap headaches and vascular spasms. However, he experienced a progressive worsening of episodic encephalopathy over a decade and developed cognitive impairment and tremors. Although no CMJ signals were found in any of the DWIs, multifocal CMJ high-signal changes, including in the paravermal area, were detected on the FLAIR images approximately when cognitive impairment developed. A subsequent genetic test revealed a GGC repeat expansion in *NOTCH2NLC*, leading to a revised diagnosis of NIID 11 years after the initial diagnosis.

Since the identification of GGC repeat expansions of *NOTCH2NLC* in 2019, NIID has rapidly emerged as the predominant cause of genetic leukoencephalopathy, mostly from the East Asian population. One Japanese study showed NIID was the most common cause (11.9%) of adult leukoencephalopathy, similar prevalence to that of Cerebral autosomal dominant arteriopathy with subcortical infarcts and leukoencephalopathy (4). A recent Chinese study also demonstrated that *NOTCH2NLC* mutation was the second most common cause of genetic leukoencephalopathy (19%) (5). However, *NOTCH2NLC* mutation has been rarely reported in European cohort (6). The incidence of NIID in adult-onset leukoencephalopathies across diverse populations remains unclear.

NIID is a neurodegenerative disease affecting nervous and non-nervous systems, which manifests diverse symptoms. Tai et al. recently proposed a clinical classification of NIID with five subtypes: cognitive impairment, movement disorder, episodic neurogenic event, autonomic dysfunction, and neuromuscular disease-dominant type (7). According to this classification, our patient with several RCVS-like attacks may be of the episodic neurogenic event-dominant type, manifested as one of acute disturbances of consciousness, encephalitic episodes, stroke-like episodes, epileptic seizures, or episodic headaches.

Patients with NIID of the episodic neurogenic event-dominant type, especially with stroke-like or encephalitic symptoms, may exhibit abnormally high signal intensities on MRI. Notably, some patients exhibit abnormal hyperintensity along the cortical and subcortical areas, similar to those in MELAS syndrome (8). These patients also show dynamic perfusion changes from initial hypoperfusion followed by subsequent hyperperfusion to chronic hypoperfusion (9). Other patients have usually demonstrated typical CMJ signal changes on DWIs along with white matter hyperintensities (WMHs) on T2-or FLAIR images (10).

However, the present patient did not show any white matter changes or typical DWI findings for almost 11 years. Moreover, he showed reduced perfusion with reversible vasoconstriction at the time of the first evaluation, and since then, various perfusion changes at each RCVS-like attack have been inconsistent with previous NIID cases. Therefore, diagnosing NIID in its early stages was challenging for this patient.

In most adult-onset NIID cases, a high signal intensity in the CMJ on DWI is a strong indicator of NIID (1). A recently published study of 223 NIID patients found that 96.6% of the patients had DWI high-intensity signals along the CMJ, which is a characteristic curvilinear lesion in most NIID cases (7). In other words, few NIID cases did not exhibit this characteristic curvilinear lesion. According to another study, DWI-negative patients were significantly younger than the patients with DWI CMJ lesions (11). Additionally, one report has described reversible lesions on DWI. The high signal intensity observed on DWI may become stronger or weaker during the course of the disease (2). In the present case, years later, only diffuse brain atrophy with multifocal high signal intensities in the CMJ were observed on FLAIR sequence, and these sequences became more apparent. Notably, the appearance of DWI abnormalities in patients with NIID may depend on the timing of imaging. Given that these focal cortical lesions on FLAIR images developed even before the DWI CMJ signal and WMHs emerged, DWI abnormalities, in this case, may have not yet appeared (11). A follow-up brain MRI examination is definitely required.

A high-signal lesion in the paravermis of the FLAIR sequence is a crucial marker for NIID, affecting 55–89% of patients with NIID (7, 12). This lesion can help clinicians distinguish NIID from fragile X–associated tremor/ataxia syndrome (FXTAS) which is clinically and pathologically similar to NIID, since the distribution of the cerebellar lesion in NIID (paravermis) differs from that observed in FXTAS (middle cerebellar peduncle) (13). Although less sensitive than CMJ lesions on DWI, paravermal lesions can take precedence over CMJ lesions and may be the only radiological sign for the diagnosis of NIID in the absence of DWI CMJ abnormalities, just like our case (14). In addition, progressive cerebral atrophy with widening of subarachnoid space and ventricles was observed on brain MRI scans performed over an 11-year period (Figure 2). The cerebral atrophy has been previously reported in patients with NIID and is believed to be a consequence of mostly astroglial and white matter pathology (2, 7, 15, 16).

The mechanisms underlying episodic neurogenic event-dominant NIID remain unclear. However, several potential hypotheses have been proposed. The first hypothesis involves the disruption of the blood–brain barrier (BBB) due to astrocytic degeneration. In affected white matter of NIID patients, reduced astrocyte density and morphological abnormalities have been observed (16). These findings may indicate a disruption of BBB, leading to impaired blood flow control. The second hypothesis suggests that blood flow dysregulation is a contributing factor. The accumulation of eosinophilic inclusions in cerebrovascular smooth muscle cells could play a significant role in vascular dysfunction, leading to altered cerebral perfusion in individuals with NIID (17). The discussion regarding whether the disruption of the BBB and blood flow dysregulation in NIID are primary or secondary changes is needed.

A limitation of this study was the absence of a skin biopsy. Recent studies have revealed that abnormal GGC amplification in *NOTCH2NLC* can also be present in Parkinson’s disease, AD, and essential tremors (18). Therefore, these overlapping findings in other neurodegenerative disorders may impact the diagnostic specificity of a GGC repeat expansion in *NOTCH2NLC*.

In conclusion, this is the first report to describe a patient with NIID who initially presented with symptoms similar with RCVS. Given that RCVS is usually benign and its pathogenesis remains unclear, clinicians need to be aware of the possibility of NIID when patients present with recurrent RCVS-like symptoms with cognitive deficits during long-term follow-up period, even if they do not show the characteristic MR findings of NIID.

## Data availability statement

The original contributions presented in the study are included in the article/Supplementary material, further inquiries can be directed to the corresponding author.

## Ethics statement

The studies involving human participants were reviewed and approved by the institutional review board of Pusan National University Hospital (2105-002-102). Written informed consent was obtained from the individual(s), and minor(s)’ legal guardian/next of kin, for the publication of any potentially identifiable images or data included in this article.

## Author contributions

G-HL: Conceptualization, Writing – original draft, Writing – review & editing. EJ: Investigation, Writing – review & editing. N-YJ: Methodology, Supervision, Writing – review & editing. TM: Methodology, Supervision, Writing – review & editing. NM: Methodology, Supervision, Writing – review & editing. E-JK: Conceptualization, Resources, Writing – review & editing, Writing – original draft.
