# Supplementary material for: Case report: Neuronal intranuclear inclusion disease initially mimicking reversible cerebral vasoconstriction syndrome: serial neuroimaging findings during an 11-year follow-up
Source: Front Neurol. 2024 Feb 9;15:1347646. doi: 10.3389/fneur.2024.1347646 (PMC10884197; doi:10.3389/fneur.2024.1347646)
Supplement: Supplementary file 1 [file Table_1.DOCX]

Supplemental table 1. Patient’s clinical manifestation, MR and CT perfusion over an an 11-years period

| Events | Age | Clinical manifestations | Duration | MRI and MRA | CT perfusion |
| --- | --- | --- | --- | --- | --- |
| 1 | 59 | Headache, confusion, visual field defect | 12 hours | No parenchymal lesion  Left P2 occlusion | 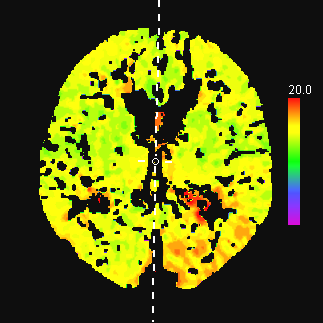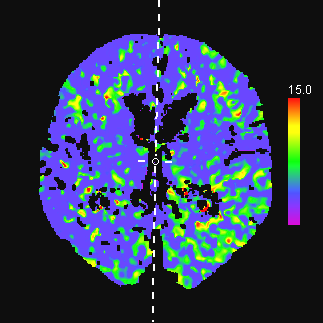  A delay (arrows) in the time-to-peak map and reduced cerebral blood flow (triangles) in the left occipital lobe |
| 2 | 60 | Confusion, visual field defect | 12 hours |  |  |
| 3 | 60 | Confusion | 12 hours |  |  |
| 4 | 62 | Headache, confusion, visual field defect | 1 day | No steno-occlusive lesion | 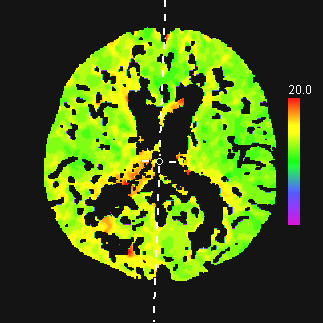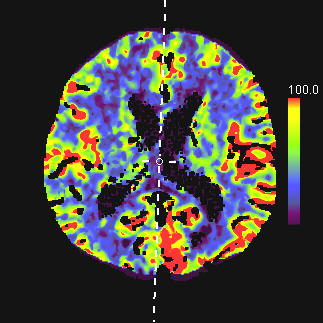 Normal |
| 5 | 63 | Headache, confusion, visual field defect | Two days | Left P2 occlusion | 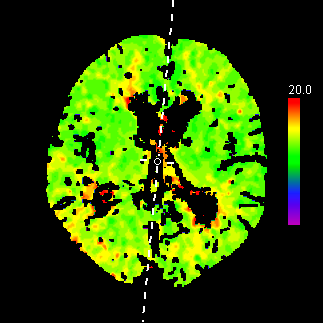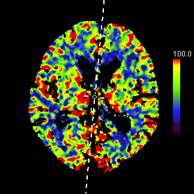 Normal |
| 6 | 64 | Syncope due to orthostatic hypotension |  |  |  |
| 7 | 65 | Headache, confusion | Two days | No steno-occlusive lesion | 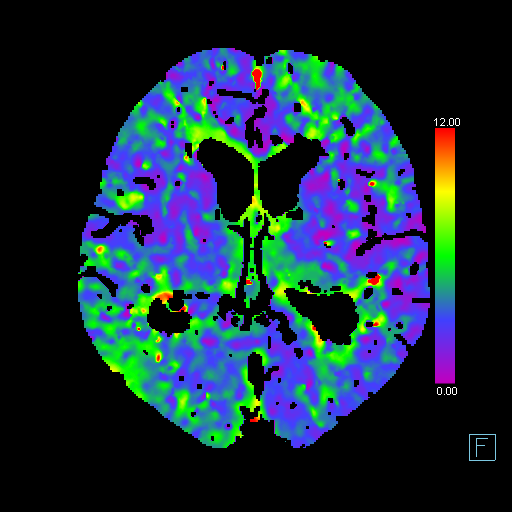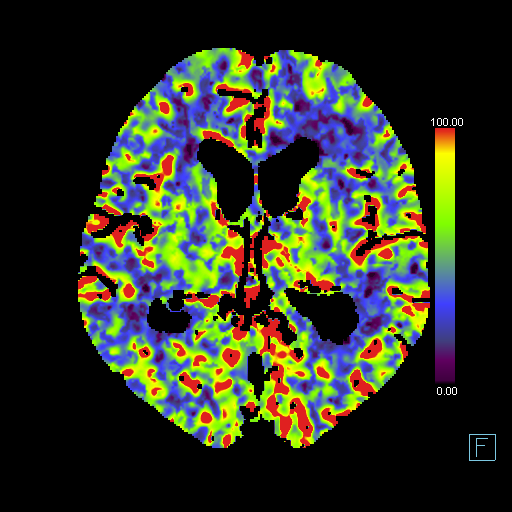 Normal |
| 8 | 66 | Headache, confusion, visual field defect | 5 days | Left P2 occlusion | 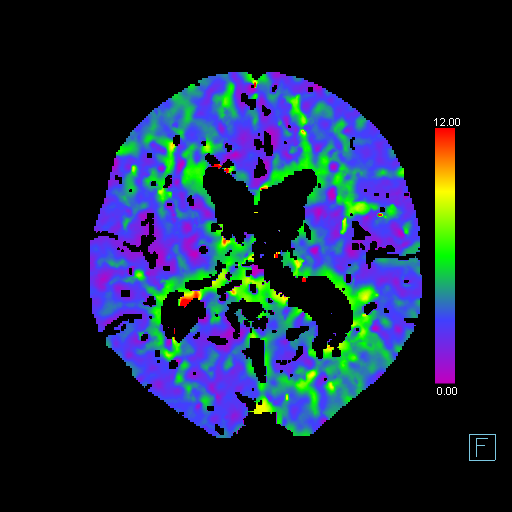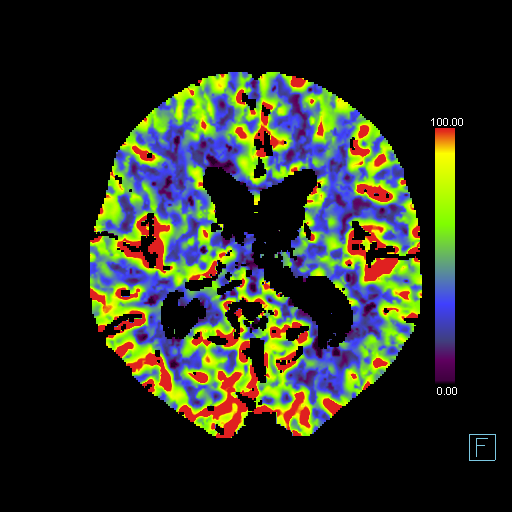 Normal |
| 9 | 66 | Seizure, visual hallucination, decreased mental status | 30 days | No steno-occlusive lesion | 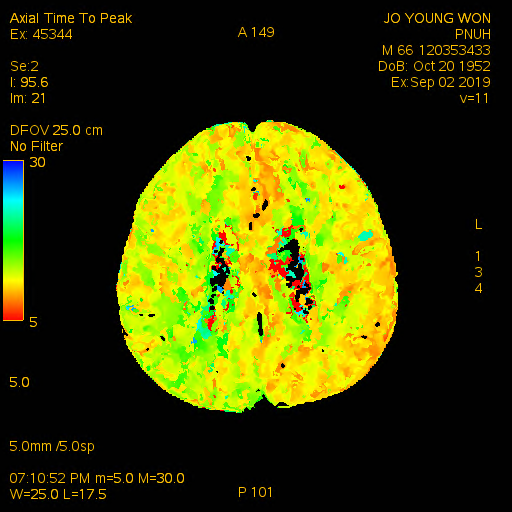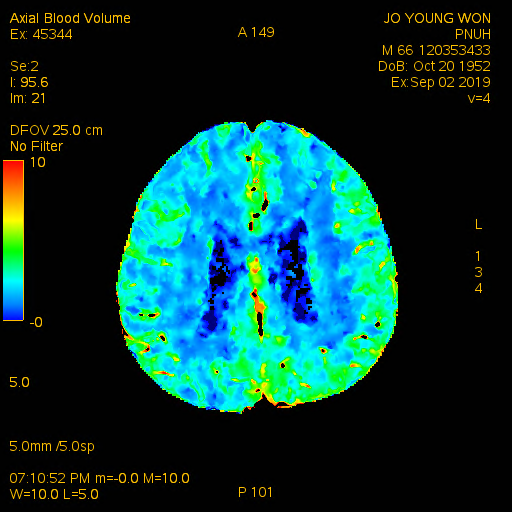  Mild delay (arrows) in time-to-peak map at the right parieto-temporal area and nearly normal cerebral blood flow. |
| 10 | 68 | Seizure, visual hallucination, decreased mental status | 28 days | No steno-occlusive lesion | 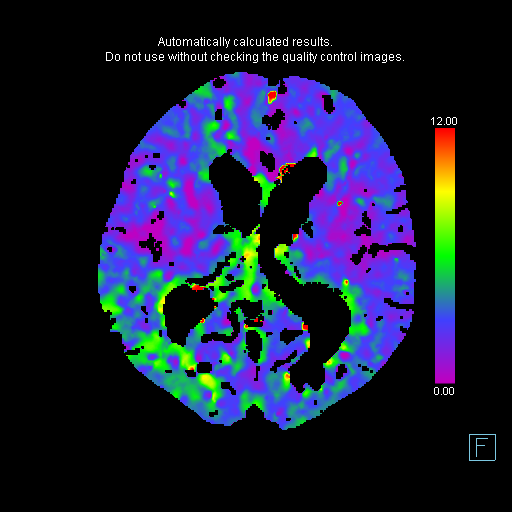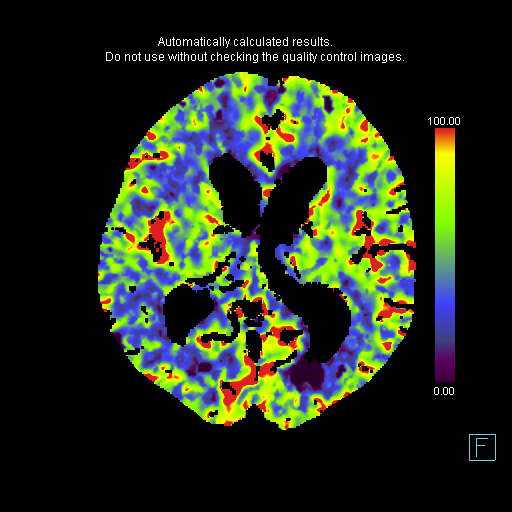  Mild delay (arrows) in time-to-peak map at the right temporo-occipital area and nearly normal cerebral blood flow |
| 11 | 70 | Right arm weakness, dysarthria | 1 hour | No steno-occlusive lesion |  |

MRI, magnetic resonance imaging; MRA, magnetic resonance angiography; CT, computed tomography
